# Body Mass Index, sex, non-steroidal anti-inflammatory drug medications, smoking and alcohol are differentially associated with World Health Organisation criteria and colorectal cancer risk in people with Serrated Polyposis Syndrome: an Australian case-control study

**DOI:** 10.1186/s12876-022-02557-7

**Published:** 2022-11-26

**Authors:** Emma Anthony, Jeanette C. Reece, Elasma Milanzi, Jihoon E. Joo, Sharelle Joseland, Mark Clendenning, Amanda Whelan, Susan Parry, Julie Arnold, Varnika Vijay, Nathan Atkinson, John L. Hopper, Aung K. Win, Mark A. Jenkins, Finlay A. Macrae, Ingrid M. Winship, Christophe Rosty, Daniel D. Buchanan, for the Australasian Coloretal Cancer Family Registry, the Family Cancer Clinics of Australia, the Genetics of Colonic Polyposis Study

**Affiliations:** 1 Department of Clinical Pathology, The University of Melbourne, Victorian Comprehensive Cancer Centre, 305 Grattan Street, Parkville, Victoria 3010 Australia; 2 University of Melbourne Centre for Cancer Research, Victorian Comprehensive Cancer Centre, Parkville, Victoria Australia; 3 Centre for Epidemiology and Biostatistics, The University of Melbourne, Carlton, Victoria Australia; 4 New Zealand Familial Gastrointestinal Cancer Service, Auckland, New Zealand; 5 Colorectal Medicine and Genetics, The Royal Melbourne Hospital, Parkville, Victoria Australia; 6 Department of Medicine, The University of Melbourne, Parkville, Victoria Australia; 7 Genomic Medicine and Family Cancer Clinic, Royal Melbourne Hospital, Parkville, Victoria Australia; 8 Envoi Pathology, Brisbane, Queensland Australia; 9 University of Queensland, School of Medicine, Herston, Queensland Australia

**Keywords:** Serrated polyposis syndrome, World Health Organization, Colorectal cancer, Case-control, Multivariate analysis, Height, Sex, Multivitamin, Folate, Calcium, NSAIDs, Medication, Cigarettes, Alcohol, Pregnancy, Hormone replacement therapy, BMI, Modifiable factors, Logistic regression

## Abstract

**Objective:**

The unknown aetiology of Serrated Polyposis Syndrome (SPS) impedes risk prediction and prevention. We investigated risk factors for SPS, overall and stratified by World Health Organization (WHO)^2010^ clinical criteria and by colorectal cancer (CRC).

**Method:**

A retrospective case-control study involving a cross-sectional analysis from 350 unrelated individuals with SPS from the Genetics of Colonic Polyposis Study and 714 controls from the Australasian Colorectal Cancer Family Registry. Univariate and multivariate logistic regression modelling was used to determine the association between risk factors and SPS and risk factors associated with CRC in SPS.

**Results:**

Female biological sex (odds ratio (OR) = 4.54; 95%Confidence interval (CI) = 2.77–7.45), increasing body mass index (BMI) at age 20 years (OR = 1.09; 95%CI = 1.04–1.13), hormone replacement therapy (OR = 0.44; 95%CI = 0.20.98), and increasing weekly folate intake (OR = 0.82; 95%CI = 0.75–0.90) were associated with SPS by multivariate analysis. Increasing weekly calcium intake (OR = 0.79; 95%CI = 0.64–0.97) and smoking > 10 cigarettes daily (OR = 0.45; 95%CI = 0.23–0.86) were associated with WHO criterion I only. The consumption of 1-100 g of alcohol per week (OR = 0.39; 95%CI = 0.18–0.83) was associated with WHO criterion III only. Smoking 1–5 cigarettes daily (OR = 2.35; 95%CI = 1.09–5.05), weekly non-steroidal anti-inflammatory drug (NSAIDs) intake (OR = 0.88; 95%CI = 0.78–0.99), and increased height (OR = 1.09; 95% = 1.05–1.13), were associated with SPS fulfilling both WHO criteria I and III. Moreover, weekly NSAIDs intake (OR = 0.81; 95%CI = 0.67–0.98) was associated with a reduced likelihood of CRC in SPS.

**Conclusion:**

We identified novel risk and potential protective factors associated with SPS, some specific for certain WHO^2010^ criteria. Weekly use of NSAIDs may reduce the risk of CRC in people with SPS.

**Supplementary Information:**

The online version contains supplementary material available at 10.1186/s12876-022-02557-7.

## Background

Serrated polyps of the large intestine are the precursor lesions of 15 to 30% of colorectal cancers (CRC) that develop via the serrated neoplasia pathway [1–3]. Serrated Polyposis Syndrome (SPS) is characterised by the occurrence of multiple serrated polyps in the colon and/or rectum and is associated with 25–50% increased risk of CRC [4–6]. Serrated polyps include hyperplastic polyps (HPs), sessile serrated lesions (SSLs; formerly known as sessile serrated adenoma/polyps (SSA/Ps)) and traditional serrated adenomas (TSAs) [7]. The prevalence of SPS is reported to be as high as 0.66% in screening populations [8]. The aetiology of SPS is largely unknown. In 2010, the World Health Organization (WHO) defined SPS using three clinical criteria (I, II and III) that were updated in 2019 [9, 10]; WHO^2010^ criterion II was abandoned, and WHO^2010^ criteria I and III were amended to WHO^2019^ criteria I and II, reflecting the two main phenotypes of SPS (Table 1) [9, 10]. It is estimated that 45% of individuals fulfill WHO^2019^ criterion I, 25% fulfill WHO^2019^ criterion II, and 30% meet both WHO^2019^ criteria I and II [10]. Although the new WHO^2019^ criteria for SPS have been revised, it has been suggested that patients who meet WHO^2010^ criteria but do not fit the WHO^2019^ criteria should still retain their SPS diagnosis [10]. For the purposes of this study, we have used the WHO^2010^ criteria to define the people with SPS in this study.

**Table 1:** Comparison of 2010 and 2019 WHO Criteria for Serrated Polyposis Syndrome (SPS)

Major SPS Phenotypes	WHO Criteria 2010	WHO Criteria 2019
**Type 1**	**I**. At least 5 serrated polyps proximal to the sigmoid Colon, 2 of which are > 10 mm in diameter.^**α**^	**I**. ≥5 Serrated lesions/polyps proximal to the rectum, all being ≥5 mm in size, with at least 2 being ≥10 mm in size
	**II**. Any number of serrated polyps occurring proximal to the sigmoid Colon in an individual who has a first-degree relative with SPS.	
**Type 2**	**III**. > 20 serrated polyps of any size distributed throughout the Colon.^**α**^	**II**. > 20 Serrated lesions/polyps of any size distributed throughout the large bowel, with ≥5 being proximal to the rectum.

^**α**^ criteria used to define SPS in this study

Recent studies have identified risk factors associated with the serrated pathway to CRC including: a family history of polyps and/or CRC, tobacco smoking, alcohol, increased body mass index (BMI), diet, diabetes mellitus, ageing, ethnicity, lack of physical activity, hormone replacement therapy (HRT), education, medications, and dietary and supplementary vitamins [11–14]. There are two previous systematic reviews and Meta-analyses on serrated lesions. A 2017 systematic review of lifestyle factors associated with serrated polyps, including HP, SSL, and/ or TSAs, found tobacco smoking, high alcohol consumption, highest red meat intake, highest fat intake and BMI ≥30 increased the risk of serrated polyps, whereas regular non-steroidal anti-inflammatory drugs (NSAIDs) or aspirin use and highest folate intake reduced risks [11]. A 2015 Meta-analysis further delineated alcohol consumption and the risk of serrated polyps and found moderate and high alcohol intake increased the risk of serrated polyps, however, this study excluded people with TSA or synchronous conventional adenomas [15].

Few studies have investigated associations between lifestyle factors or patient characteristics, stratified by respective WHO SPS criteria, especially studies investigating participants who meet both criteria I^2010^ and III^2010^ [16–20]. Moreover, these studies only examined limited risk factors including sex, smoking and CRC or polyp-affected first-degree relatives and were limited by study size and specific WHO criteria examined. The 2016 cohort study by Petronio et al. found that people with WHO^2010^ SPS criterion I were mostly women and there was a high prevalence of smokers, however, only 29 people were examined [17]. The large 2017 retrospective Dutch and British multicentre study involving 434 people with SPS found that only tobacco smoking had an inverse association with CRC in people with SPS, however, risk factors associated with SPS were not stratified by WHO criteria [16].

The present study aimed to investigate the association between environmental and lifestyle exposures and SPS and to determine whether these risk factors differed by SPS WHO criteria, using data from the Genetics of Colonic Polyposis Study [9]. We also investigated which risk factors were associated with the development of CRC in SPS.

## Methods

### Study Population

Prospectively collected case data were obtained from the Genetic Colonic Polyposis Study (GCPS). Participants who met the WHO^2010^ criteria I and/or III for SPS were recruited into the GCPS between 2007 and 2019 from gastroenterologists and Family Cancer Clinics across Australia. All proband participants who met WHO criteria I and/or III for SPS, regardless of any family history of polyps or cancer, diagnosed with SPS ≥18 years of age and who had completed the structured questionnaire were included in the study (*n =* 350). Participants with SPS were excluded if they fulfilled only the WHO^2010^ SPS criterion II, carried a germline pathogenic variant in one of the DNA mismatch repair genes or in one of the other known genetic predispositions to CRC or had incomplete information on serrated polyp counts and WHO criteria. Information on risk factor exposures prior to SPS diagnosis from GCPS participants were collected through a self-reported questionnaire during recruitment and clinicopathological information was collected from colonoscopy and pathology reports and pedigree information.

Controls were selected to provide 2:1 ratio of controls to cases to increase study power and were comprised of 714 participants; 266 population-based participants recruited into the Australasian Colorectal Cancer Family Registry (ACCFR) between 1996 and 2008 from the Australian electoral roll and 448 spouses of CRC-affected probands recruited into the ACCFR between 1997 and 2012. Inclusion criteria of controls included no personal history of CRC. Risk factor data for ACCFR participants were collected through a self-reported questionnaire and pedigree information. Use of data from GCPS and ACCFR for this study were approved by the Human Research Ethics Committee of the University of Melbourne (#1442811 and #1954921, respectively).

### Data Collection

Self-reported information on demographics, personal characteristics, medical history, reproduction, diet, tobacco smoking, alcohol intake, current body weight, body weight at 20 years of age and height was obtained from all cases in GCPS and controls in ACCFR [21]. SPS diagnosis and CRC diagnoses were verified for each case using pathology reports, medical records and cancer registry reports. CRC was defined as any primary diagnosis of invasive adenocarcinoma in the colon (ICD-O-3 codes C18.0, C18.2–C18.9 and C26.0), rectosigmoid junction (C19.9) or rectum (C20.9 and C21.8) [22].

### Data Preparation

Outcomes assessed were: 1) SPS, 2) SPS stratified by WHO criteria I, III and meeting both criteria, and 3) SPS stratified by the presence or absence of CRC. The sample population for outcome 2 excluded SPS participants with missing polyp sizes in their pathology reports, as we could not determine if they fulfilled only criterion III or both criteria. Exposures of interest were sex, smoking, height, BMI, diabetes, medication, supplements and alcohol consumption. Matching controls to cases by age was considered, however, it was decided it would not be beneficial as unmatched cases would have been excluded from the analyses, resulting in reduced sample size and thus a decrease in power to test the hypotheses [23–25]. Furthermore, there would be a loss of efficiency for adjusting for confounding in the logistic regression models when restricting the analyses to a subset of cases, especially with a low matching ratio. Weekly medication and supplement intake were captured during the time of first diagnosis for cases and during the time when they were regularly taking medications or supplements for controls. Alcohol variables for cases were observed during the 5 years leading up to the time of first diagnosis and for controls it was dependent on the age bracket they completed the questionnaire (20s, 30s–40s or 50s+). Wine, spirits and beer were converted to grams of ethanol (14 g of ethanol in 12-oz serving of beer, 11.2 g in 4-oz serving of wine, and 9.3 g in 1-oz serving of spirits). This was calculated using Brick’s (2006) standardization of alcohol calculations in research and converting 1-oz equivalent to 29.57 ml [26]. Grams of alcohol for wine, spirits and beer were then combined to create total weekly alcohol consumption. Categorization of alcohol was based on a 2018 combined analysis for risk thresholds for alcohol consumption [27]. Female only exposures were also investigated, which included the number of pregnancies, age of first pregnancy; and HRT use. Variables that could not be harmonized or had greater than 20% missing data were excluded from the analyses; this included other chronic disease types, diabetes, oral contraception, iron supplements, and weekly coffee and tea consumption. BMI at the time of the questionnaire and BMI at the age of 20 years was derived respectively from current weight, and weight at the age of 20 years, thus weight was excluded from the models due to multicollinearity. ‘Current smoking’ and ‘smoking ever’ variables were combined to create ‘smoking status’, which was recoded into one variable with three categories. Further, the variable daily cigarettes was recoded into an ordinal categorical variable.

### Statistical Analysis

Descriptive statistics were used to explore the data. Univariate and multivariate logistic models were used to quantify the association. Firstly, univariate analyses were performed for all exposures of interest. Exposures with less than 20% missing data and a *P-*value< 0.15 in univariate analyses were included in multivariate analyses. The 20% was arbitrarily chosen to minimize missing observations for the multivariate model, which would decrease the power and precision of the estimates. Similarly, *P-*value of < 0.15 was an arbitrary choice but it is a conventional technique used to choose predictor variables for multivariate logistic regression models [28]. Smoking variables and the ordinal categorical variable observing weekly alcohol consumption were included in the multivariate models regardless of the *P-*values from the univariate model, as these were exposures of interest. The main multivariate logistic regression model included smoking status as an ordinal categorical variable and alcohol type as separate continuous variables. Separate multivariate models were fitted to provide estimates for different versions of smoking and alcohol variables. As some exposures, such as number of pregnancies, were specific to women, univariate and multivariate Sub-analyses were performed for females only. Multiple comparison corrections were not necessary as SPS (yes/no) is the primary analysis and the stratified analyses by WHO criteria and CRC, are considered secondary analyses [29]. Results were presented as odds ratios (ORs) with 95% confidence intervals (CI), and *P-*values < 0.05 were considered statistically significant. All analyses were performed in STATA version 15 [30].

## Results

A total of 350 people with SPS and 714 controls were included in the case-control analyses (Table 2). For people with SPS, 150 (43%) fulfilled WHO criterion I, 67 (19%) fulfilled WHO criterion III, 109 (31%) met both WHO criteria I and III for SPS. Twenty-four people with SPS (7%) fulfilled WHO criterion III but were missing details on polyp size to determine if they also fulfilled both criteria, so were excluded from stratified analyses. SPS cases had a greater proportion of females (65%) and were of a younger age (median diagnosis age of 39 years, interquartile range (IQR) of 29–57 years) compared to controls, which comprised 57% females and a median age of 50 years (IQR = 44–55) at the time of completing the questionnaire. Twenty-three percent (82/350) of the people with SPS developed CRC with an average age at CRC diagnosis of 53 years (IQR = 37–66) (Tables 2 and 5).

**Table 2:** Associations between lifestyle risk factors and SPS

Characteristics	Sample Size	Controls (*N =* 714)	SPS (*N =* 350)	Univariate Analysis	Multivariate Analysis	
		*N (%)*	*N* (%)	*P*-value	*OR (95% CI)*	*P*-value
Sex (Female)	1064	404 (57)	227 (65)	0.010	**4.54 (2.77–7.45)**	**< 0.001**
Age/ Diagnosis Age (years) (median, IQR)	1051	39 (29–57)	50 (44–55)	< 0.001	**0.96 (0.95–0.98)**	**< 0.001**
Affected with CRC	1064	0 (0)	82 (23)	–		
Smoking Status	1039					
Never Smoked	504	334 (47)	170 (52)	Ref.		
Current Smoker	152	120 (17)	32 (10)	0.003	**0.50 (0.29–0.86)**	**0.012**
Former Smoker	383	258 (36)	125 (38)	0.732	1.30 (0.90–1.88)	0.156
Years Smoked (mean ± S.D.)	1036	10.20 ± 12.43	9.78 ± 14.34	0.631	1.01 (0.99–1.02)	0.456
Daily Cigarettes	1038					
No Cigarettes	505	334 (47)	171 (52)	Ref.		
1–5 Cigarettes per day	99	54 (8)	45 (14)	0.029	1.52 (0.87–2.65)	0.139
6–10 Cigarettes per day	112	78 (11)	34 (10)	0.477	0.74 (0.42–1.31)	0.306
> 10 Cigarettes per day	322	246 (35)	76 (23)	0.002	0.70 (0.46–1.05)	0.085
Height (cm) (mean ± S.D.)	946	168.61 ± 10.11	171 .08 ± 9.38	< 0.001	**1.07 (1.04–1.09)**	**< 0.001**
Weight (kg) (mean ± S.D.)	951	75.56 ± 16.94	76.71 ± 15.90	–		
Weight at 20 years of age (kg) (mean ± S.D.)	935	63.22 ± 12.91	68.35 ± 14.43	–		
BMI at registration (mean ± S.D.)	940	26.51 ± 5.29	26.25 ± 5.13	0.483		
BMI at 20 years of age (mean ± S.D.)	924	22.14 ± 3.53	23.46 ± 4.73	< 0.001	**1.09 (1.04–1.13)**	**< 0.001**
Diabetes	1027	31 (4)	15 (5)	0.749		
Blood Lipid Lowering Medication	1007	65 (9)	36 (12)	0.241		
Weekly Aspirin (dose/week) (mean ± S.D.)	1024	0.99 ± 3.36	0.36 ± 1.61	< 0.001	0.95 (0.88–1.02)	0.153
Weekly NSAIDs (dose/week) (mean ± S.D.)	1014	1.72 ± 4.62	0.50 ± 2.27	< 0.001	**0.91 (0.86–0.97)**	**0.002**
Weekly Antacids (dose/week) (mean ± S.D.)	1023	1.20 ± 4.75	0.88 ± 4.63	0.291		
Weekly Multivitamins (dose/week) (mean ± S.D.)	1022	1.79 ± 3.44	1.18 ± 2.65	0.004	0.94 (0.89–1.00)	0.053
Calcium (dose/week) (mean ± S.D.)	1021	1.01 ± 2.70	0.48 ± 2.02	0.001	0.97 (0.89–1.05)	0.395
Paracetamol (dose/week) (mean ± S.D.)	1017	1.86 ± 7.60	1.54 ± 5.33	0.501		
Folate (dose/week) (mean ± S.D.)	1015	1.09 ± 2.53	0.31 ± 1.54	< 0.001	**0.82 (0.75–0.90)**	**< 0.001**
Weekly Alcohol Consumption	903					
No Alcohol	266	171 (27)	95 (35)	Ref.		
1–100 g per week	388	285 (46)	103 (37)	0.012	**0.56 (0.37–0.84)**	**0.005**
101–200 g per week	150	100 (16)	50 (18)	0.625	0.99 (0.59–1.67)	0.984
201–350 g per week	63	46 (7)	17 (6)	0.190	0.67 (0.31–1.44)	0.307
> 350 g per week	36	25 (4)	11 (4)	0.543	0.52 (0.20–1.36)	0.183
Beer (serves per week) (mean ± S.D.)	919	1.78 ± 5.44	2.29 ± 6.33	0.232		
Wine (serves per week) (mean ± S.D.)	918	4.09 ± 6.34	2.93 ± 5.97	0.006	0.97 (0.94–1.00)	0.089
Spirits (serves per week) (mean ± S.D.)	925	1.37 ± 4.34	0.88 ± 3.03	0.060	0.96 (0.91–1.01)	0.093
Number of Pregnancies^a^ (mean ± S.D.)	603	2.67 ± 1.61	1.80 ± 1.75	< 0.001	0.87 (0.75–1.01)	0.069
Pregnancy Age (years) ^a^ (mean ± S.D.)	489	25.68 ± 4.86	25.49 ± 5.06	0.700		
Hormone Replacement Therapy (HRT)^a^	547	74 (21)	14 (7)	< 0.001	**0.44 (0.20–0.98)**	**0.043**

In the multivariate models, it was multivariate adjusted. Each group of cases is compared with controls. Variables were included in the multivariate model if it had a *p-*value of < 0.15 and less than < 20% missing data. A variable was considered significant in the multivariate model if it had a *p-*value < 0.05. For variables that were highly correlated, the smaller *p-*value was selected as the proxy to be included in the multivariate model.

^a^ female only analysis

Results from the multivariate logistic model assessing risk factors of SPS showed a higher BMI at 20 years of age was associated with SPS, with 9% (95%CI = 1.04–1.13; *p <* 0.001) increased odds of developing SPS for every 1 kg/cm^2^ increase in BMI at 20 years of age compared with controls. Taller participants were associated with 7% increased odds of SPS, with 8% increased odds found in the female only subgroup analysis (Table 2 and Supplementary Table 1). Conversely, increasing weekly folate and NSAIDs intake decreased the odds of SPS by 18% (95%CI = 0.75–0.90; *p <* 0.001) and 9% (95%CI = 0.86–0.97; *p =* 0.002), respectively (Table 2). In a female subgroup analysis, HRT supplements was associated with a 56% decreased odds of SPS (95%CI = 0.20–0.98; *p =* 0.043) compared with not taking HRT (Table 2 and Supplementary Table 1).

### Risk Factors by WHO^2010^ Criteria I and III

After multivariate adjustment, female biological sex was associated with 2.14-fold (95% CI = 1.26–3.62; *p =* 0.005) increased odds of fulfilling WHO criterion I and 5.74-fold (95% CI = 2.72–12.10; *p <* 0.001) increased odds of fulfilling both WHO I and III criteria compared with males (Tables 3 and 4). Increasing height (OR = 1.09; 95%CI = 1.05–1.13; *p <* 0.001) and smoking 1–5 cigarettes per day compared with those that never smoked increased the odds (OR = 2.35, 95%CI = 1.09–5.05; *p =* 0.029) of fulfilling both WHO criteria. Conversely, current smokers and smoking greater than 10 cigarettes per day had a decreased odds of fulfilling WHO criterion I by 0.16-fold (95% CI = 0.16; *p =* 0.001) and 0.45-fold (95%CI = 0.23–0.86; *p* = 0.015), respectively (Table 3).

**Table 3:** Associations between lifestyle risk factors and those who exclusively fulfil either WHO criterion I or III for SPS

Characteristics	Controls (*N =* 714)	SPS WHO Criteria I (*N =* 150)	Univariate Analysis	Multivariate Analysis		SPS WHO Criteria III (*N =* 67)	Univariate Analysis	Multivariate Analysis	
	*N (%)*	*N* (%)	*P-*value	*OR (95% CI)*	*P-*value	*N (%)*	*P-*value	*OR (95% CI)*	*P-*value
Sex (Female)	404 (57)	103 (69)	0.006	**2.14 (1.26–3.62)**	**0.005**	39 (58)	0.797		
Age/ Diagnosis Age (years) (median, IQR)	50 (44–55)	36.5 (29–54)	< 0.001	**0.93 (0.91–0.96)**	**< 0.001**	48 (29–59)	< 0.001	**0.95 (0.92–0.97)**	**< 0.001**
Affected with CRC	0 (0)	33 (22)	–			18 (27)	–		
Smoking Status									
Never Smoked	334 (47)	83 (61)				24 (36)	Ref.		
Current Smoker	121 (17)	5 (4)	< 0.001	**0.16 (0.06–0.49)**	**0.001**	16 (24)	0.073	0.90 (0.37–2.17)	0.817
Former Smoker	258 (36)	47 (35)	0.121	1.03 (0.61–1.75)	0.915	26 (40)	0.252	1.31 (0.67–2.56)	0.432
Years Smoked (mean ± S.D.)	10.20 ± 12.43	6.96 ± 12.06	0.004	0.99 (0.97–1.01)	0.323	14.49 ± 16.83	0.013	1.02 (0.99–1.04)	0.220
Daily Cigarettes									
No Cigarettes	334 (47)	83 (62)				25 (39)	Ref.		
1–5 Cigarettes per day	54 (8)	14 (10)	0.896	1.02 (0.45–2.32)	0.964	9 (14)	0.054	2.22 (0.87–5.68)	0.094
6–10 Cigarettes per day	78 (11)	14 (10)	0.302	0.66 (0.28–1.53)	0.332	4 (6)	0.494	0.84 (0.27–2.61)	0.769
> 10 Cigarettes per day	246 (35)	23 (17)	< 0.001	**0.45 (0.23–0.86)**	**0.015**	26 (41)	0.238	0.92 (0.44–1.90)	0.817
Height (cm) (mean ± S.D.)	168.61 ± 10.11	169.46 ± 9.14	0.389			170.71 ± 9.38	0.004	1.03 (1.00–1.06)	0.050
Weight (kg) (mean ± S.D.)	75.56 ± 16.94	75.21 ± 16.36	0.832	–		76.01 ± 15.97	–		
Weight at 20 years of age (kg) (mean ± S.D.)	63.22 ± 12.91	68.59 ± 15.07	0.001	–		69.64 ± 15.51	–		
BMI at registration (mean ± S.D.)	26.51 ± 5.30	26.37 ± 5.83	0.794			26.89 ± 4.90	0.608		
BMI at 20 years of age (mean ± S.D.)	22.14 ± 3.53	23.68 ± 5.36	< 0.001	**1.06 (1.00–1.13)**	**0.044**	23.53 ± 4.67	0.012	**1.08 (1.00–1.16)**	**0.043**
Diabetes	31 (4)	5 (4)	0.820			1 (2)	0.272		
Blood Lipid Lowering Medication	65 (9)	9 (7)	0.411			7 (12)	0.502		
Weekly Aspirin (dose/week) (mean ± S.D.)	0.99 ± 3.36	0.17 ± 1.07	< 0.001	0.88 (0.73–1.06)	0.187	0.49 ± 1.69	0.193		
Weekly NSAIDs (dose/week) (mean ± S.D.)	1.72 ± 4.62	0.42 ± 1.62	< 0.001	0.90 (0.81–1.00)	0.060	0.75 ± 2.90	0.075	0.94 (0.86–1.04)	0.224
Weekly Antacids (dose/week) (mean ± S.D.)	1.20 ± 4.75	0.42 ± 1.84	0.022	0.88 (0.73–1.02)	0.077	1.31 ± 3.26	0.867		
Weekly Multivitamins (dose/week) (mean ± S.D.)	1.79 ± 3.44	0.83 ± 2.10	< 0.001	0.93 (0.85–1.03)	0.152	1.29 ± 2.93	0.259		
Calcium (dose/week) (mean ± S.D.)	1.01 ± 2.70	0.17 ± 1.07	< 0.001	**0.79 (0.64–0.97)**	**0.021**	0.55 ± 1.81	0.164		
Paracetamol (dose/week) (mean ± S.D.)	1.86 ± 7.60	0.77 ± 3.88	0.067	0.99 (0.94–1.04)	0.749	1.75 ± 4.96	0.919		
Folate (dose/week) (mean ± S.D.)	1.09 ± 2.53	0.37 ± 1.78	< 0.001	**0.84 (0.73–0.96)**	**0.010**	0.36 ± 1.56	0.016	0.88 (0.74–1.06)	0.177
Weekly Alcohol Consumption									
No Alcohol	171 (27)	38 (34)	Ref.			21 (40)	Ref.		
1–100 g per week	285 (46)	45 (40)	0.156	0.87 (0.49–1.56)	0.640	15 (28)	0.016	**0.39 (0.18–0.83)**	**0.015**
101–200 g per week	100 (16)	22 (20)	0973	1.75 (0.83–3.71)	0.144	7 (13)	0.216	0.51 (0.19–1.37)	0.185
201–350 g per week	46 (7)	4 (3)	0.089	0.39 (0.08–1.87)	0.238	6 (11)	0.902	0.75 (0.23–2.38)	0.622
> 350 g per week	25 (4)	3 (3)	0.333	0.75 (0.18–3.20)	0.702	4 (8)	0.652	0.66 (0.16–2.66)	0.516
Beer (serves per week) (mean ± S.D.)	1.78 ± 5.44	1.77 ± 5.31	0.978			3.11 ± 7.16	0.145	1.01 (0.97–1.06)	0.554
Wine (serves per week) (mean ± S.D.)	4.09 ± 6.34	2.61 ± 3.99	0.008	0.98 (0.94–1.04)	0.541	4.20 ± 10.30	0.905		
Spirits (serves per week) (mean ± S.D.)	1.37 ± 4.34	0.73 ± 2.27	0.067	0.90 (0.80–1.02)	0.106	0.84 ± 3.17	0.305		
Number of Pregnancies (mean ± S.D.)^a^	2.67 ± 1.61	1.74 ± 1.90	< 0.001	0.90 (0.70–1.15)	0.397	1.94 ± 1.50	0.009	0.96 (0.69–1.34)	0.821
Pregnancy Age (years) (mean ± S.D.) ^a^	25.68 ± 4.86	27.15 ± 5.03	0.034	1.03 (0.96–1.11)	0.415	25.12 ± 5.23	0.578		
Hormone Replacement Therapy (HRT) ^a^	74 (21)	5 (6)	< 0.001	0.47 (0.15–1.52)	0.207	3 (10)	0.151		

In the multivariate models, it was multivariate adjusted. Each group of cases is compared with controls. Variables were included in the multivariate model if it had a *p-*value of < 0.15 and less than < 20% missing data. A variable was considered significant in the multivariate model if it had a *p-*value < 0.05. For variables that were highly correlated the smaller *p-*value was selected as the proxy to be included into the multivariate model.

^a^ female only analysis

**Table 4:** Associations between lifestyle risk factors and those who meet the both WHO criteria I and III for SPS

Characteristics	Controls (*N =* 714)	Both Criteria (*N =* 109)	Univariate Analysis both Criteria	Multivariate Analysis	
	*N (%)*	*N (%)*	*P-*value	*OR (95% CI)*	*P-*value
Sex (Female)	404 (57)	70 (64)	0.130	**5.74 (2.72–12.10)**	**< 0.001**
Age/ Diagnosis Age (years) (median, IQR)	50 (44–55)	36 (30–57.5)	< 0.001	**0.95 (0.92–0.97)**	**< 0.001**
Affected with CRC	0 (0)	24 (22)	–		
Smoking Status					
Never Smoked	334 (47)	46 (43)	Ref.		
Current Smoker	121 (17)	12 (11)	0.233	0.80 (0.36–1.78)	0.587
Former Smoker	258 (36)	50 (46)	0.428	1.68 (0.96–2.93)	0.069
Years Smoked (mean ± S.D.)	10.20 ± 12.43	9.45 ± 13.83	0.568	1.01 (0.99–1.04)	0.306
Daily Cigarettes					
No Cigarettes	334 (47)	51 (49)	Ref.		
1–5 Cigarettes per day	54 (8)	20 (19)	0.003	**2.35 (1.09–5.05)**	**0.029**
6–10 Cigarettes per day	78 (11)	15 (14)	0.470	0.92 (0.41–2.12)	0.863
> 10 Cigarettes per day	246 (35)	19 (18)	0.016	0.67 (0.34–1.30)	0.236
Height (cm) (mean ± S.D.)	168.61 ± 10.11	172.24 ± 9.62	0.001	**1.09 (1.05–1.13)**	**< 0.001**
Weight (kg) (mean ± S.D.)	75.56 ± 16.94	76.90 ± 15.59	–		
Weight at 20 years of age (kg) (mean ± S.D.)	63.22 ± 12.91	68.28 ± 12.95	–		
BMI at registration (mean ± S.D.)	26.51 ± 5.30	25.80 ± 4.33	0.193		
BMI at 20 years of age (mean ± S.D.)	22.14 ± 3.53	22.99 ± 3.63	0.037	1.06 (0.99–1.13)	0.122
Diabetes	31 (4)	7 (7)	0.295		
Blood Lipid Lowering Medication	65 (9)	14 (14)	0.148	**3.36 (1.36–8.33)**	**0.009**
Weekly Aspirin (dose/week) (mean ± S.D.)	0.99 ± 3.36	0.53 ± 2.08	0.130	0.96 (0.86–1.07)	0.450
Weekly NSAIDs (dose/week) (mean ± S.D.)	1.72 ± 4.62	0.36 ± 1.85	< 0.001	**0.88 (0.78–0.99)**	**0.028**
Weekly Antacids (dose/week) (mean ± S.D.)	1.20 ± 4.75	1.33 ± 7.37	0.812		
Weekly Multivitamins (dose/week) (mean ± S.D.)	1.79 ± 3.44	1.49 ± 2.99	0.376		
Calcium (dose/week) (mean ± S.D.)	1.01 ± 2.70	0.78 ± 2.84	0.393		
Paracetamol (dose/week) (mean ± S.D.)	1.86 ± 7.60	2.14 ± 6.68	0.730		
Folate (dose/week) (mean ± S.D.)	1.09 ± 2.53	0.27 ± 1.37	< 0.001	**0.82 (0.70–0.96)**	**0.012**
Weekly Alcohol Consumption					
No Alcohol	171 (27)	28 (31)	Ref.		
1–100 g per week	285 (46)	33 (37)	0.207	**0.39 (0.20–0.75)**	**0.005**
101–200 g per week	100 (16)	21 (23)	0.429	1.09 (0.51–2.34)	0.821
201–350 g per week	46 (7)	5 (6)	0.425	0.54 (0.16–1.82)	0.322
> 350 g per week	25 (4)	3 (3)	0.629	0.16 (0.02–1.43)	0.102
Beer (serves per week) (mean ± S.D.)	1.78 ± 5.44	2.40 ± 6.55	0.437		
Wine (serves per week) (mean ± S.D.)	4.09 ± 6.34	2.68 ± 4.81	0.025	0.95 (0.90–1.00)	0.067
Spirits (serves per week) (mean ± S.D.)	1.37 ± 4.34	1.18 ± 3.92	0.680		
Number of Pregnancies (mean ± S.D.)^a^	2.67 ± 1.61	1.58 ± 1.53	< 0.001	**0.75 (0.57–0.98)**	**0.035**
Pregnancy Age (years) (mean ± S.D.) ^a^	25.68 ± 4.86	24.81 ± 4.26	0.260		
Hormone Replacement Therapy (HRT) ^a^	74 (21)	5 (8)	0.009	0.33 (0.08–1.32)	0.116

In the multivariate models, it was multivariate adjusted. Each group of cases is compared with controls. Variables were included in the multivariate model if it had a *p-*value of < 0.15 and less than < 20% missing data. A variable was considered significant in the multivariate model if it had a *p-*value < 0.05. For variables that were highly correlated the smaller *p-*value was selected as the proxy to be included into the multivariate model.

^a^ female only analysis

The results for medications and supplement intake showed variable effects between the different WHO criteria. Use of calcium and folate was associated with decreased odds of fulfilling WHO criterion I by 21% (95% CI = 0.64–0.97; *p =* 0.021) and 16% (95% CI = 0.73–0.96; *p =* 0.010), respectively, for every extra dose taken per week. While weekly NSAIDs and folate intake were associated with a decreased odds for meeting both WHO criteria I and III by 12% (95%CI = 0.78–0.99; *p =* 0.028) and 18% (95% CI = 0.70–0.96; *p =* 0.012), respectively (Table 4). Blood lipid lowering medication increased the odds of fulfilling both criteria by 3.36-fold (95%CI = 1.36–8.33; *p =* 0.009). Further, 1–100 g of alcohol consumption per week decreased the odds of WHO criterion III and those that fulfilled both criteria by 61% (WHO criterion III: 95%CI = 0.18–0.83, *p =* 0.015, both WHO criteria: 95%CI = 0.20–0.75, *p =* 0.005).

**Fig. 1:**
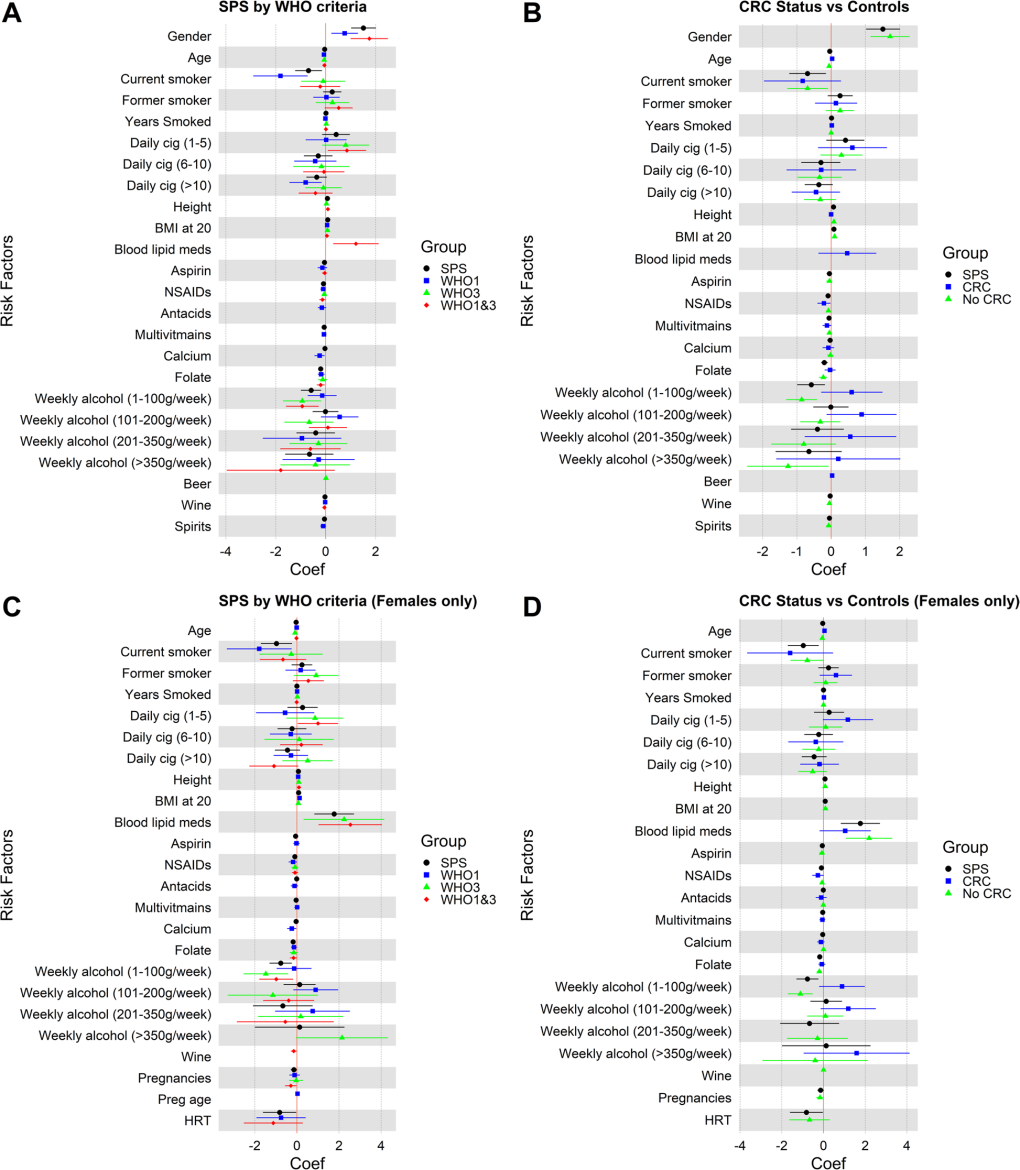
Forest plots summarizing odds ratio (OR) and 95% confidence intervals (CI) for each characteristic and risk factor. **A** OR and 95% CI for characteristics and risk factors for all SPS (black dots) and stratified by SPS WHO criteria (WHO^2010^ criteria I = blue square; WHO^2010^ criteria III = green triangle; Both WHO^2010^ criteria I and III = red diamond) vs. controls **B** OR and 95% CI for characteristics and risk factors for all SPS (black dots) and stratified by SPS CRC status (developed CRC = blue square; did not develop CRC = green triangle) vs. controls. **C** OR and 95% CI for characteristics and risk factors for all SPS (black dots) and stratified by SPS WHO criteria (WHO^2010^ criteria I = blue square; WHO^2010^ criteria III = green triangle; Both WHO^2010^ criteria I and III = red diamond) vs. controls for females only. **D** OR and 95% CI for characteristics and risk factors for all SPS (black dots) and stratified by SPS CRC status (developed CRC = blue square; did not develop CRC = green triangle) vs. controls for females only. Variables were excluded from the figure if they were not included in the multivariate model (*p-*value of > 0.15 and > 20% missing data in the univariate analysis)

For females, there was a 25% (95%CI = 0.57–0.98; *p =* 0.035) decreased odds of fulfilling both WHO criteria for every one-year increase in age at first pregnancy. However, no hormonal factors were associated with those that fulfilled either criterion I only or criterion III only (Supplementary Table 2 and Supplementary Table 3). Notably, blood lipid lowering medication had a greater magnitude for females that fulfilled WHO criterion III and both criteria, after controlling for sex (Supplementary Table 3 and Supplementary Table 4). Females had a 9.36-fold (95% CI = 1.38–63.33; *p =* 0.022) increased odds of fulfilling WHO criterion III (Supplementary Table 3) and 12.62-fold (95% CI = 2.81–56.64; *p =* 0.001) of fulfilling both criteria (Supplementary Table 3) if they were taking blood lipid lowering medication; while no associations were found for males (univariate model: WHO criterion III *p =* 0.755 and both WHO criteria *p =* 1.000). Forest plots summarizing OR and 95% CI for each characteristic and risk factor stratified by WHO criteria vs. controls is shown in Fig. 1 A and for females only in Fig. 1 C.

### Risk Factors by CRC Development

Of the 350 participants with SPS, 82 (23.4%) were diagnosed with CRC by the time of enrollment. Both criterion I (33/150) and participants that fulfilled WHO criteria I and III (24/109) had equivalent CRC prevalence at 22% whilst criterion III was higher at 27%.

Examining the association between exposures and SPS, with or without CRC, in separate multivariate models found only age and NSAID intake had an association with participants with CRC and SPS (Table 5). Every extra NSAID dose per week compared with controls was associated with a 19% reduction in the odds of CRC (95%CI = 0.67–0.98; *p =* 0.031), while every 1-year increase in age increased the odds of CRC by 3% (95%CI = 1.00–1.07; *p =* 0.039) compared to controls. Neither sex or smoking showed any significant associations with CRC in people with SPS, and no associations were found between blood lipid lowering medication and hormonal factors in women and CRC (Table 5 and Supplementary Table 5). In contrast to associations found in the WHO criteria stratified analyses, SPS patients without CRC had an inverse association with high alcohol intake (> 350 g/week) and wine intake. For those consuming greater than 350 g of alcohol per week, there was a 72% (95%CI = 0.09–0.94; *p =* 0.039) decrease in the odds of SPS without CRC compared to no alcohol intake, and every increase in serving of wine consumed per week decreased the odds of SPS without CRC by 5% (95%CI = 0.91–0.99; *p =* 0.016)(Table 5). Results from the females only analysis are shown in Supplementary Table 6. Forest plots summarizing OR and 95% CI for each characteristic and risk factor stratified by CRC status vs. controls is shown in Fig. 1 B and for females only in Fig. 1 D.

**Table 5:** Associations between lifestyle risk factors and CRC status

Characteristics	Controls (*N =* 714)	SPS Cases with CRC (*N =* 82)	Univariate Analysis	Multivariate Analysis		SPS Cases with No CRC (*N =* 267)	Univariate Analysis	Multivariate Analysis	
	*N (%)*	*N (%)*	*P-*value	*OR (95% CI)*	*P-*value	*N (%)*	*P-*value	*OR (95% CI)*	*P-*value
Sex (Female)	404 (57)	46 (56)	0.933			180 (67)	0.002	**5.58 (3.16–9.86)**	**< 0.001**
Age/ Diagnosis Age (median, IQR.)	50 (44–55)	53 (37–66)	0.005	**1.03 (1.00–1.07)**	**0.039**	35 (29–54)	< 0.001	**0.94 (0.93–0.96)**	**< 0.001**
Affected with CRC	0 (0)	82 (100)	–			0			
Smoking Status									
Never Smoked	334 (47)	35 (47)	Ref.			127 (49)	Ref.		
Current Smoker	121 (17)	9 (12)	0.378	0.44 (0.14–1.34)	0.147	31 (12)	0.082	**0.50 (0.28–0.91)**	**0.024**
Former Smoker	258 (36)	31 (41)	0.599	1.15 (0.62–2.13)	0.653	101 (39)	0.853	1.30 (0.86–1.96)	0216
Years Smoked (mean ± S.D.)	10.20 ± 12.43	14.11 ± 17.82	0.018	1.02 (1.00–1.04)	0.114	8.54 ± 12.92	0.069	1.00 (0.99–1.02)	0.836
Daily Cigarettes									
No Cigarettes	334 (47)	37 (50)	Ref.			133 (53)			
1–5 Cigarettes per day	54 (8)	7 (10)	0.719	1.86 (0.68–5.09)	0.225	38 (15)	0.016	1.35 (0.73–2.48)	0.340
6–10 Cigarettes per day	78 (11)	7 (10)	0.625	0.75 (0.27–2.07)	0.580	27 (11)	0.569	0.71 (0.37–1.36)	0.304
> 10 Cigarettes per day	246 (35)	22 (30)	0.448	0.64 (0.32–1.30)	0.219	54 (21)	0.001	0.72 (0.45–1.15)	0.175
Height (cm) (mean ± S.D.)	168.61 ± 10.11	170.73 ± 10.29	0.123	1.00 (0.96–1.03)	0.752	171.20 ± 9.17	0.001	**1.08 (1.05–1.11)**	**< 0.001**
Weight (kg) (mean ± S.D.)	75.56 ± 16.94	75.95 ± 13.13	–			76.86 ± 16.57			
Weight at 20 years of age (kg) (mean ± S.D.)	63.22 ± 12.91	63.30 ± 9.87	–			69.59 ± 15.12			
BMI at registration (mean ± S.D.)	26.51 ± 5.30	26.15 ± 4.58	0.608			26.25 ± 5.27	0.523		
BMI at 20 years of age (mean ± S.D.)	22.14 ± 3.53	21.80 ± 2.55	0.485			23.85 ± 5.04	< 0.001	**1.10 (1.05–1.16)**	**< 0.001**
Diabetes	31 (4)	3 (5)	0.877			12 (5)	0.755		
Blood Lipid Lowering Medication	65 (9)	10 (16)	0.116	1.61 (0.69–3.74)	0.270	26 (11)	0.524		
Weekly Aspirin (dose/week) (mean ± S.D.)	0.99 ± 3.36	0.67 ± 2.07	0.414			0.28 ± 1.46	< 0.001	0.95 (0.87–1.03)	0.230
Weekly NSAIDs (dose/week) (mean ± S.D.)	1.72 ± 4.62	0.25 ± 1.25	0.001	**0.81 (0.67–0.98)**	**0.031**	0.56 ± 2.45	< 0.001	**0.92 (0.87–0.98)**	**0.013**
Weekly Antacids (dose/week) (mean ± S.D.)	1.20 ± 4.75	0.78 ± 3.11	0.441			0.91 ± 4.96	0.384		
Weekly Multivitamins (dose/week) (mean ± S.D.)	1.79 ± 3.44	0.83 ± 2.23	0.015	0.89 (0.78–1.01)	0.071	1.28 ± 2.75	0.029	0.95 (0.89–1.02)	0.160
Calcium (dose/week) (mean ± S.D.)	1.01 ± 2.70	0.35 ± 1.52	0.030	0.93 (0.78–1.10)	0.382	0.51 ± 2.14	0.005	0.98 (0.90–1.07)	0.678
Paracetamol (dose/week) (mean ± S.D.)	1.86 ± 7.60	2.85 ± 7.53	0.367			1.22 ± 4.59	0.191		
Folate (dose/week) (mean ± S.D.)	1.09 ± 2.53	0.44 ± 1.72	0.029	0.97 (0.82–1.14)	0.685	0.28 ± 1.49	< 0.001	**0.79 (0.71–0.89)**	**< 0.001**
Weekly Alcohol Consumption									
No Alcohol	171 (27)	9 (16)	Ref.			86 (39)	Ref.		
1–100 g per week	285 (46)	24 (42)	0.243	1.83 (0.75–4.48)	0.187	79 (36)	0.001	**0.42 (0.27–0.67)**	**< 0.001**
101–200 g per week	100 (16)	13 (23)	0.045	2.44 (0.88–6.77)	0.088	36 (17)	0.155	0.73 (0.41–1.31)	0.287
201–350 g per week	46 (7)	7 (12)	0.045	1.76 (0.46–6.67)	0.407	10 (5)	0.025	0.45 (0.18–1.15)	0.094
> 350 g per week	25 (4)	4 (7)	0.081	1.23 (0.20–7.54)	0.820	7 (3)	0.191	**0.28 (0.09–0.94)**	**0.039**
Beer (serves per week) (mean ± S.D.)	1.78 ± 5.44	3.97 ± 9.33	0.024	1.03 (1.00–1.07)	0.083	1.91 ± 5.22	0.852		
Wine (serves per week) (mean ± S.D.)	4.09 ± 6.34	4.95 ± 6.06	0.340			2.43 ± 5.86	< 0.001	**0.95 (0.91–0.99)**	**0.016**
Spirits (serves per week) (mean ± S.D.)	1.37 ± 4.34	0.93 ± 3.11	0.398			0.82 ± 2.93	0.046	0.94 (0.87–1.00)	0.062
Number of Pregnancies (mean ± S.D.)^a^	2.67 ± 1.61	2.74 ± 2.41	0.820			1.60 ± 1.51	< 0.001	0.85 (0.71–1.02)	0.096
Pregnancy Age (years) (mean ± S.D.) ^a^	25.68 ± 4.86	25.32 ± 4.98	0.695			25.50 ± 5.11	0.734		
Hormone Replacement Therapy (HRT) ^a^	74 (21)	4 (13)	0.244			10 (6)	< 0.001	0.49 (0.19–1.29)	0.147

In the multivariate models, it was multivariate adjusted. Each group of cases is compared with controls. Variables were included in the multivariate model if it had a *p-*value of < 0.15 and less than < 20% missing data. A variable was considered significant in the multivariate model if it had a *p-*value < 0.05. For variables that were highly correlated the smaller *p-*value was selected as the proxy to be included into the multivariate model.

^a^ female only analysis

## Discussion

### Environmental and lifestyle exposures associated with SPS WHO criteria^2010^ I and III

In this case-control study of 350 people with SPS and 714 controls, we identified several factors associated with a reduced risk of SPS that have not previously been reported, some of which were specific for certain WHO criteria. Folate intake and NSAIDs reduced the risk of SPS by 18 and 9%, respectively. When stratifying risks by WHO criteria, increasing calcium and folate intake decreased the odds of fulfilling WHO criterion I by 21 and 16%, respectively. Similarly, folate and NSAIDs intake reduced the risk of fulfilling both SPS WHO criteria by 18 and 12%, respectively. Of interest, 1–100 g of alcohol per week compared to no alcohol was associated with a reduced risk of fulfilling WHO criterion III and both criteria, and participants that fulfilled WHO criterion I were less likely to smoke > 10 cigarettes a day. Higher BMI when 20 years old (OR = 1.09), being taller (OR = 1.07), and female biological sex (OR = 4.54 and specifically, OR = 2.14 for criterion I and OR = 5.74 for both criteria) had increased risk of SPS, respectively. Use of blood lipid lowering medication in women was strongly associated with fulfilling both WHO criteria (OR = 12.62).

Height was positively associated with SPS in our study, consistent with a postulated effect on cancer development due to the association with a person’s height and the number of body cells, genetic make-up, and exposure to hormone and growth factors during developmental stages [31]. Height was also identified in a 2018 Meta-analysis of three prospective case-control studies as a risk factor for serrated polyps [32]. We observed that BMI was also positively associated with SPS risk. This is consistent with two Meta-analyses studies that found high BMI, high triglyceride levels and high triglyceride to high-density lipoprotein cholesterol (TG/ HDL) ratios were associated with an increased risk of serrated polyps, especially in the distal Colon [11, 33–36]. Drew et al. found that for every 1 unit increase in BMI, the risk of serrated polyps increased by 2% [37]. Collectively, these findings suggest obesity, and high cholesterol and triglyceride levels may be risk factors for serrated polyp development including for the development of multiple serrated polyps as seen in SPS. This finding may be particularly relevant for women who meet both WHO criteria I and III for SPS, due to the association also found with blood lipid lowering medication in the present study (OR = 12.62).

We found that being female was associated with SPS. Consistent with these findings, previous studies also found sex was associated with serrated polyps and SPS [3, 14, 16, 38, 39]. In the female-only analyses, we found advancing primigravida age was associated with a decreased odds of fulfilling both WHO criteria by 25% and HRT use decreased the odds of developing SPS by 56%, but no associations were found with WHO criterion I alone. In a clinic-based case-control study, Morimoto et al. also found HRT to have an inverse association with hyperplastic polyps, however, no associations were found between parity or age of first live pregnancy and hyperplastic polyps [40]. A prospective cohort study of 594 cases with serrated polyps, found cases were less likely to have estrogen-only HRT than controls [35]. Collectively, these findings suggest hormonal factors such as HRT may be protective against the serrated pathway and could improve the clinical management of women with SPS.

Our study found further evidence to support the potentially protective effect of vitamin supplements on SPS. Folate intake appeared protective against both SPS phenotypes, and calcium supplementation was only protective against the criteria I phenotype. Consistent with these findings, the 2017 Bailie et al. systematic review of 43 studies found high intake of folate and calcium, decreased the risk of serrated polyps [11]. In contrast, a previous randomized control trial found calcium supplementation increased the risk of developing SSL after 6–10 years of intake [41]. We found that NSAIDs had a protective effect for participants fulfilling both SPS WHO criteria. These findings are consistent with the Bailie et al. systematic review and a more recent case-control study of 214 people with SSL and 560 with HP, both of which found high NSAIDs intake reduced the risk of serrated polyps [11, 42].

The role of cigarette exposure to SPS has been evaluated in several studies, which reported smokers were more likely to have distal, left-sided colorectal serrated polyps than non-smokers [35, 36, 43], consistent with the increased risk of fulfilling both WHO criteria found in our study. Other studies found strong associations between cigarette smoking and serrated polyps [36, 44, 45], with the case-control study of 40–70 year old participants reporting a positive association with increasing daily cigarettes and all polyps, and > 10 cigarettes increasing the odds of distal serrated polyps by 5.58-fold (95%CI = 2.33–13.35; *p <* 0.001) [45]. Further, one case-control study found SPS patients were more likely to be current smokers than controls [46]. Conversely, one of the largest SPS cohort studies of 434 people with SPS found smoking was associated with a 63% reduction in risk of CRC [16]. We found a similar inverse association between SPS and smoking. Greater than 10 cigarettes per day was inversely associated with WHO criterion I, whilst 1–5 cigarettes per day was positively associated with both criteria. This present study differentiated by amount of smoking exposure and found differing effects on SPS behavior. The conflicting findings with smoking exposure warrant further investigations through prospective studies.

### Exposures associated with the development of CRC in people with SPS

Twenty three percent of people with SPS in our study had CRC; 27% with WHO criterion III, 22% with WHO criterion I and 22% with both criteria I and III. Supporting our findings, previous estimates of the prevalence of CRC in those with serrated polyps was 20–30%, as reported by a 2011 US study and two European multicenter cohort studies [16, 47, 48]. A recent 2021 Meta-analysis also found the overall risk of CRC in 2788 patients with SPS was 20% [49]. The European multicenter study also found participants fulfilling both WHO^2010^ criteria I and III were associated with the highest CRC risk [16]. Although we found a higher prevalence of CRC in participants that met only WHO criterion III in our study, this was not significantly different to the other WHO criteria groups studied. Further, in the present study, females were not at increased risk of developing CRC compared with males.

When examining potential risk factors associated with the development of CRC in SPS patients, we found NSAIDs was associated with a 19% reduction in the risk of developing CRC for every extra dose taken per week. These findings support the Tsioulias et al. (2015) [50] literature review examining the effects of NSAIDs on CRC, where prolonged use of NSAIDs reduced the incident of CRC by 30–50%. However, the cumulative toxicity of NSAIDs needed to be considered if NSAIDs are to be used as a chemoprevention tool [50]. Two randomized, double-blind, placebo-controlled trials of patients with familial adenomatous polyps (FAP) also found a type of NSAID, Sulindac, effectively reduced the size and number of colorectal polyps [51, 52]. Collectively, these studies suggest the benefits of NSAIDs is not limited to the adenoma-carcinoma pathway; and depending on dosage and duration, may be beneficial for people with SPS to help reduce the risk of CRC.

Of note, low to moderate alcohol intake (1–100 g per week OR = 0.53), and drinking wine appeared to reduce the risk of SPS diagnosis compared to abstainers. Controversially, high amounts of alcohol (> 350 g) was also associated with reduced risk of SPS, though for patients who developed CRC these associations did not hold. Although not specific for participants with SPS, two previous systematic reviews found small to moderate consumption of wine was potentially protective against CRC in individuals of average risk compared with no wine consumption [53, 54]. A 2016 German population-based study found protective effects on the survival of CRC-affected patients with small consumption of wine [55]. However, in contrast to our study, two previous prospective studies found current moderate to heavy alcohol increased the odds of HP and CRC in the distal colon [56, 57], with a further retrospective case-control study of 132 HP cases identifying alcohol as a risk factor for the association between HP and CRC [58].

### Strengths

Strengths of this study included the relatively large sample size of 350 participants with SPS and the comprehensive list of variables examined. This enabled robust statistical analyses to be performed to investigate the association between multiple risk factors, including the clinical outcome of CRC development, and different SPS WHO criteria, which has not been extensively reported in the literature. Selection bias was also limited as we clearly defined the study population with strict inclusion and exclusion criteria. Further, the case-control design of the study increased the external validity of study findings. In order to avoid differential bias, the GCPS and ACCFR datasets were harmonized to ensure only questionnaire data that was the same between datasets or could be derived from the dataset was used in analyses.

### Limitations

As some demographic or exposure variable data was missing due to participants not completing self-reported questionnaires, variables with ≥20% missing data were excluded from our analysis. For GCPS participants, servings per week for medication and supplements were asked in the questionnaire, however, exact dosage in one tablet was not specified. Further research would be needed to discover if the association changed for a higher or lower dosage of medication or supplementation. A small subset of people with SPS (7%) who fulfilled WHO criterion III were missing details on polyp size and, therefore, could not be excluded from fulfilling both criteria so were excluded from stratified analyses. Family history of CRC and polyps was not assessed in this study as 67% of the controls were spouses of CRC-affected participants. There was a low percentage of ACCFR controls that had baseline colonoscopies (27% of population and 27% of spousal controls). While we could not completely preclude the possibility of SPS among controls who never underwent colonoscopy, we believe this would have minimally influenced our results as the frequency of SPS in screening populations is very low [8]. Further, since this present study was cross-sectional, temporality was not observed. Subsequently, causality could not be established. Likewise, the progression of events could not be estimated, as there could be reverse causality. Further prospective studies could be used to address whether risk factors and characteristics associated with WHO criteria for SPS are causal or associated due to unknown confounders.

## Conclusion

Previously, there has been a paucity of evidence examining the effect modifiable factors may have on the risk of developing SPS. This present study provided new evidence of the potentially protective role that folate, calcium and NSAIDs use, low to moderate alcohol intake and HRT may have on the development of SPS and on the development of CRC in SPS patients. However, the retrospective nature of this study limits causality for these modifiable risk factors and more studies are needed before these protective factors can be recommended as interventions to decrease the risk of developing different SPS phenotypes.

Further, factors that may be potential risk indicators for developing SPS include high BMI at 20 years of age, blood lipid lowering medication, increasing height and female gender. These findings, combined with findings from other studies that identified BMI, blood lipid lowering medication and female biological sex as risk factors for serrated polyps, could assist with risk stratification for prevention and screening strategies specific for patients with different SPS phenotypes. Incorporation of these modifiable factors into current risk stratification algorithms for SPS management could help reduce colonoscopy burden in those at lower risk of CRC, whilst increasing attention to those at greater risk of developing CRC.

## Supplementary Information

**Additional file 1: Supplementary Table 1.** Female Sub-analysis investigating the association between SPS and characteristics/ lifestyle factors. **Supplementary Table 2.** Female Sub-analysis investigating the association between WHO criteria I and characteristics/ lifestyle factors. **Supplementary Table 3.** Female Sub-analysis investigating the association between WHO criteria III and characteristics/ lifestyle factors. **Supplementary Table 4.** Female Sub-analysis investigating the association between both WHO criteria I and III and characteristics/ lifestyle factors. **Supplementary Table 5. **Female Sub-analysis investigating the association between CRC and characteristics/ lifestyle factors. **Supplementary Table 6.** Female Sub-analysis investigating the association between SPS patients with no CRC and characteristics/ lifestyle factors

## Data Availability

The datasets used and/or analysed during the current study are available from the corresponding author on reasonable request.
