# Supplementary material for: Body Mass Index, sex, non-steroidal anti-inflammatory drug medications, smoking and alcohol are differentially associated with World Health Organisation criteria and colorectal cancer risk in people with Serrated Polyposis Syndrome: an Australian case-control study
Source: BMC Gastroenterol. 2022 Nov 26;22:489. doi: 10.1186/s12876-022-02557-7 (PMC9701413; doi:10.1186/s12876-022-02557-7)
Supplement: Supplementary file 1 — Additional file 1: Supplementary Table 1. Female Sub-analysis investigating the association between SPS and characteristics/ lifestyle factors. Supplementary Table 2. Female Sub-analysis investigating the association between WHO criteria I and characteristics/ lifestyle factors. Supplementary Table 3. Female Sub-analysis investigating the association between WHO criteria III and characteristics/ lifestyle factors. Supplementary Table 4. Female Sub-analysis investigating the association between both WHO criteria I and III and characteristics/ lifestyle factors. Supplementary Table 5. Female Sub-analysis investigating the association between CRC and characteristics/ lifestyle factors. Supplementary Table 6. Female Sub-analysis investigating the association between SPS patients with no CRC and characteristics/ lifestyle factors [file 12876_2022_2557_MOESM1_ESM.docx]

**Supplementary Tables**

**Supplementary Table 1**: Female sub-analysis investigating the association between SPS and characteristics/ lifestyle factors.

| Characteristics | Sample Size | Controls (*N=*404) | Total SPS WHO Criteria (*N=*227) | Univariate Analysis | Multivariate Analysis | |
| --- | --- | --- | --- | --- | --- | --- |
|  |  | *N (%)* | *N* (%) | *P-*value | *OR (95% CI)* | *P-*value |
| Age/ Diagnosis Age (years) (median, IQR) | 624 | 50 (43-54) | 36 (29-56) | <0.001 | **0.96 (0.94-0.99)** | **0.001** |
| Affected with CRC |  | 0 (0) | 46 (20) | - | | |
| Smoking Status | 621 |  | | | | |
| Never Smoked | 322 | 204 (51) | 118 (54) | Ref. | | |
| Current Smoker | 98 | 74 (18) | 24 (11) | 0.027 | **0.38 (0.18-0.80)** | **0.011** |
| Former Smoker | 201 | 126 (31) | 75 (35) | 0.878 | 1.27 (0.78-2.07) | 0.341 |
| Years Smoked (mean ± S.D.) | 613 | 9.08 ± 11.89 | 8.57 ± 13.55 | 0.630 | 1.00 (0.98-1.02) | 0.720 |
| Daily Cigarettes | 617 |  | | | | |
| No Cigarettes | 327 | 204 (51) | 123 (58) | Ref. | | |
| 1-5 Cigarettes per day | 67 | 38 (9) | 29 (14) | 0.386 | 1.31 (0.64-2.69) | 0.457 |
| 6-10 Cigarettes per day | 75 | 53 (13) | 22 (10) | 0.180 | 0.79 (0.40-1.57) | 0.504 |
| > 10 Cigarettes per day | 148 | 109 (27) | 39 (18) | 0.017 | 0.64 (0.35-1.16) | 0.139 |
| Height (cm) (mean ± S.D.) | 557 | 162.54 ± 7.30 | 166.78 ± 7.46 | <0.001 | **1.08 (1.04-1.11)** | **<0.001** |
| Weight (kg) (mean ± S.D.) | 561 | 69.06 ± 16.00 | 72.47 ± 14.85 | **-** | | |
| Weight at 20 (kg) (mean ± S.D.) | 546 | 57.21 ± 11.01 | 64.38 ± 14.30 | **-** | | |
| BMI at registration (mean ± S.D.) | 552 | 26.15 ± 5.91 | 26.11 ± 5.60 | 0.895 |  |  |
| BMI at 20 (mean ± S.D.) | 543 | 21.63 ± 3.74 | 23.27 ± 5.45 | <0.001 | **1.09 (1.03-1.15)** | **0.002** |
| Diabetes | 609 | 12 (3) | 8 (4) | 0.547 |  |  |
| Blood Lipid Lowering Medication | 595 | 20 (5) | 24 (12) | 0.003 | **5.89 (2.30-15.03)** | **<0.001** |
| Aspirin (dose/week) (mean ± S.D.) | 606 | 0.76 ± 3.23 | 0.33 ± 1.62 | 0.046 | 0.94 (0.85-1.05) | 0.275 |
| NSAIDs (dose/week) (mean ± S.D.) | 605 | 1.81 ± 4.82 | 0.52 ± 2.36 | <0.001 | **0.91 (0.84-0.99)** | **0.022** |
| Antacids (dose/week) (mean ± S.D.) | 607 | 1.34 ± 5.34 | 0.62 ± 2.18 | 0.036 | 0.99 (0.91-1.07) | 0.750 |
| Multivitamins (dose/week) (mean ± S.D.) | 607 | 1.97 ± 3.67 | 1.45 ± 2.92 | 0.071 | 0.96 (0.89-1.04) | 0.339 |
| Calcium (dose/week) (mean ± S.D.) | 606 | 1.63 ± 3.23 | 0.72 ± 2.46 | <0.001 | 0.96 (0.87-1.06) | 0.404 |
| Paracetamol (dose/week) (mean ± S.D.) | 603 | 2.14 ± 8.82 | 1.72 ± 5.66 | 0.529 |  |  |
| Folate (dose/week) (mean ± S.D.) | 600 | 1.83 ± 3.06 | 0.47 ± 1.88 | <0.001 | **0.83 (0.75-0.92)** | **<0.001** |
| Weekly Alcohol Consumption | 535 |  | | | | |
| No Alcohol | 189 | 115 (33) | 74 (40) | Ref. | | |
| 1 – 100 g per week | 250 | 176 (50) | 74 (40) | 0.036 | **0.46 (0.27-0.78)** | **0.004** |
| 101 – 200 g per week | 71 | 43 (12) | 28 (15) | 0.967 | 1.13 (0.53-2.45) | 0.740 |
| 201 – 350 g per week | 20 | 14 (4) | 6 (3) | 0.426 | 0.51 (0.12-2.11) | 0.353 |
| >350 g per week | 5 | 3 (1) | 2 (1) | 0.969 | 1.14 (0.14-9.54) | 0.902 |
| Beer (serves per week) (mean ± S.D.) | 549 | 0.32 ± 1.85 | 0.54 ± 1.83 | 0.172 |  |  |
| Wine (serves per week) (mean ± S.D.) | 539 | 3.39 ± 5.05 | 2.86 ± 5.77 | 0.251 |  |  |
| Spirits (serves per week) (mean ± S.D.) | 547 | 1.25 ± 3.43 | 0.99 ± 3.44 | 0.386 |  |  |
| Number of Pregnancies (mean ± S.D.) | 603 | 2.67 ± 1.61 | 1.80 ± 1.75 | <0.001 | 0.87 (0.75-1.01) | 0.069 |
| Pregnancy Age (years) (mean ± S.D.) | 489 | 25.68 ± 4.86 | 25.49 ± 5.06 | 0.700 |  |  |
| Hormone Replacement Therapy | 547 | 74 (21) | 14 (7) | <0.001 | **0.44 (0.20-0.98)** | **0.043** |

**Supplementary Table 2**: Female sub-analysis investigating the association between WHO criteria I and characteristics/ lifestyle factors.

| Characteristics | Sample Size | Controls (*N=*404) | SPS WHO Criteria 1 (*N=*103) | Univariate Analysis | Multivariate Analysis | |
| --- | --- | --- | --- | --- | --- | --- |
|  |  | *N (%)* | *N* (%) | *­P-value* | *OR (95% CI)* | *P-value* |
| Age/ Diagnosis Age (years) (median, IQR) | 500 | 50 (43-54) | 36 (29-52) | <0.001 | 0.99 (0.96-1.03) | 0.676 |
| Affected with CRC | 506 | 0 (0) | 21 (21) | - | | |
| Smoking Status | 498 |  | | | | |
| Never Smoked | 263 | 204 (51) | 59 (63) | Ref. | | |
| Current Smoker | 79 | 74 (18) | 5 (5) | 0.003 | **0.17 (0.04-0.78)** | **0.022** |
| Former Smoker | 156 | 126 (31) | 30 (30) | 0.439 | 1.20 (0.59-2.44) | 0.625 |
| Years Smoked (mean ± S.D.) | 494 | 9.08 ± 11.89 | 6.95 ± 12.62 | 0.115 | 1.01 (0.98-1.04) | 0.509 |
| Daily Cigarettes | 496 |  | | | | |
| No Cigarettes | 264 | 204 (51) | 60 (65) | - | | |
| 1-5 Cigarettes per day | 47 | 38 (9) | 9 (10) | 0.587 | 0.57 (0.14-2.28) | 0.430 |
| 6-10 Cigarettes per day | 62 | 53 (13) | 9 (10) | 0.158 | 0.75 (0.28-2.02) | 0.568 |
| > 10 Cigarettes per day | 123 | 109 (27) | 14 (15) | 0.010 | 0.75 (0.33-1.71) | 0.498 |
| Height (cm) (mean ± S.D.) | 449 | 162.54 ± 7.30 | 165.65 ± 6.66 | <0.001 | **1.06 (1.01-1.13)** | **0.020** |
| Weight (kg) (mean ± S.D.) | 447 | 69.06 ± 16.00 | 72.90 ± 15.17 | - | | |
| Weight at 20 (kg) (mean ± S.D.) | 442 | 57.21 ± 11.01 | 64.24 ± 14.93 | - | | |
| BMI at registration (mean ± S.D.) | 444 | 26.17 ± 5.88 | 26.43 ± 6.18 | 0.815 |  |  |
| BMI at 20 (mean ± S.D.) | 441 | 21.65 ± 3.72 | 23.58 ± 6.08 | 0.001 | **1.13 (1.04-1.23)** | **0.004** |
| Diabetes | 493 | 12 (3) | 3 (3) | 0.844 |  |  |
| Blood Lipid Lowering Medication | 482 | 20 (5) | 7 (8) | 0.310 |  |  |
| Aspirin (dose/week) (mean ± S.D.) | 491 | 0.76 ± 3.23 | 0.24 ± 1.28 | 0.065 | 0.98 (0.84-1.13) | 0.769 |
| NSAIDs (dose/week) (mean ± S.D.) | 489 | 1.81 ± 4.82 | 0.35 ± 1.39 | <0.001 | 0.83 (0.68-1.02) | 0.076 |
| Antacids (dose/week) (mean ± S.D.) | 492 | 1.34 ± 5.34 | 0.44 ± 1.95 | 0.051 | 0.89 (0.75-1.07) | 0.214 |
| Multivitamins (dose/week) (mean ± S.D.) | 491 | 1.97 ± 3.67 | 0.97 ± 2.29 | 0.007 | 1.01 (0.89-1.14) | 0.901 |
| Calcium (dose/week) (mean ± S.D.) | 490 | 1.63 ± 3.23 | 0.25 ± 1.29 | <0.001 | **0.78 (0.63-0.97)** | **0.026** |
| Paracetamol (dose/week) (mean ± S.D.) | 490 | 2.14 ± 8.82 | 1.02 ± 4.57 | 0.195 |  |  |
| Folate (dose/week) (mean ± S.D.) | 484 | 1.83 ± 3.06 | 0.53 ± 2.12 | <0.001 | **0.87 (0.76-1.00)** | **0.049** |
| Weekly Alcohol Consumption | 430 |  | | | | |
| No Alcohol | 145 | 115 (33) | 30 (38) | Ref. | | |
| 1 – 100 g per week | 208 | 176 (50) | 32 (40) | 0.199 | 0.88 (0.38-2.00) | 0.752 |
| 101 – 200 g per week | 58 | 43 (12) | 15 (19) | 0.424 | 2.44 (0.84-7.07) | 0.100 |
| 201 – 350 g per week | 16 | 14 (4) | 2 (3) | 0.442 | 2.11 (0.36-12.45) | 0.409 |
| >350 g per week | 3 | 3 (1) | 0 (0) | - | 1 | - |
| Beer (serves per week) (mean ± S.D.) | 437 | 0.32 ± 1.85 | 0.42 ± 1.48 | 0.633 |  |  |
| Wine (serves per week) (mean ± S.D.) | 432 | 3.39 ± 5.05 | 2.93 ± 4.36 | 0.430 |  |  |
| Spirits (serves per week) (mean ± S.D.) | 436 | 1.25 ± 3.43 | 0.87 ± 2.47 | 0.302 |  |  |
| Number of Pregnancies (mean ± S.D.) | 491 | 2.67 ± 1.61 | 1.74 ± 1.90 | <0.001 | 0.90 (0.70-1.15) | 0.397 |
| Pregnancy Age (years) (mean ± S.D.) | 408 | 25.68 ± 4.86 | 27.15 ± 5.03 | 0.034 | 1.03 (0.96-1.11) | 0.415 |
| Hormone Replacement Therapy | 443 | 74 (21) | 5 (6) | <0.001 | 0.47 (0.15-1.52) | 0.207 |

**Supplementary Table 3**: Female sub-analysis investigating the association between WHO criteria III and characteristics/ lifestyle factors

| Characteristics | Sample Size | Controls (*N=*404) | SPS WHO Criteria 3 (*N=*39) | Univariate Analysis | Multivariate Analysis | |
| --- | --- | --- | --- | --- | --- | --- |
|  |  | *N (%)* | *N (%)* | *­P-value* | *OR (95% CI)* | *P-value* |
| Age/ Diagnosis Age (years) (median, IQR) | 443 | 50 (43-54) | 36 (26-59) | <0.001 | **0.91 (0.87-0.96)** | **<0.001** |
| Affected with CRC | 443 | 0 (0) | 8 (21) |  | | |
| Smoking Status | 443 |  | | | | |
| Never Smoked | 221 | 204 (51) | 17 (44) | Ref. | | |
| Current Smoker | 83 | 74 (18) | 9 (23) | 0.384 | 0.76 (0.17-3.43) | 0.723 |
| Former Smoker | 139 | 126 (31) | 13 (33) | 0.580 | 2.48 (0.86-7.18) | 0.094 |
| Years Smoked (mean ± S.D.) | 440 | 9.08 ± 11.89 | 11.81 ± 14.75 | 0.198 | 1.02 (0.98-1.07) | 0.346 |
| Daily Cigarettes | 443 |  | | | | |
| No Cigarettes | 222 | 204 (51) | 18 (46) | Ref. | | |
| 1-5 Cigarettes per day | 43 | 38 (9) | 5 (13) | 0.456 | 2.37 (0.61-9.13) | 0.211 |
| 6-10 Cigarettes per day | 56 | 53 (13) | 3 (8) | 0.490 | 1.11 (0.21-5.76) | 0.899 |
| > 10 Cigarettes per day | 122 | 109 (27) | 13 (33) | 0.431 | 1.67 (0.50-5.55) | 0.402 |
| Height (cm) (mean ± S.D.) | 395 | 162.54 ± 7.30 | 167.71 ± 7.44 | <0.001 | **1.09 (1.02-1.17)** | **0.010** |
| Weight (kg) (mean ± S.D.) | 393 | 69.06 ± 16.00 | 76.15 ± 15.18 | - | | |
| Weight at 20 (kg) (mean ± S.D.) | 390 | 57.21 ± 11.01 | 65.44 ± 16.86 | - | | |
| BMI at registration (mean ± S.D.) | 391 | 26.17 ± 5.89 | 27.41 ± 5.93 | 0.282 |  |  |
| BMI at 20 (mean ± S.D.) | 390 | 21.65 ± 3.73 | 23.64 ±5.83 | 0.015 | 1.02 (0.98-1.18) | 0.150 |
| Diabetes | 438 | 12 (3) | 1 (3) | 0.992 |  |  |
| Blood Lipid Lowering Medication | 427 | 20 (5) | 4 (12) | 0.135 | **9.36 (1.38-63.33)** | **0.022** |
| Aspirin (dose/week) (mean ± S.D.) | 437 | 0.76 ± 3.23 | 0.32 ± 1.36 | 0.344 |  |  |
| NSAIDs (dose/week) (mean ± S.D.) | 435 | 1.81 ± 4.82 | 0.50 ± 2.44 | 0.053 | 0.92 (0.78-1.09) | 0.311 |
| Antacids (dose/week) (mean ± S.D.) | 438 | 1.34 ± 5.34 | 1.41 ± 3.23 | 0.938 |  |  |
| Multivitamins (dose/week) (mean ± S.D.) | 437 | 1.97 ± 3.67 | 2.21 ± 3.57 | 0.726 |  |  |
| Calcium (dose/week) (mean ± S.D.) | 437 | 1.63 ± 3.23 | 0.88 ± 2.29 | 0.155 |  |  |
| Paracetamol (dose/week) (mean ± S.D.) | 436 | 2.14 ± 8.82 | 2.79 ± 6.26 | 0.693 |  |  |
| Folate (dose/week) (mean ± S.D.) | 430 | 1.83 ± 3.06 | 0.62 ± 2.02 | 0.013 | 0.86 (0.71-1.05) | 0.151 |
| Weekly Alcohol Consumption | 382 |  | | | | |
| No Alcohol | 132 | 115 (33) | 17 (55) | Ref. | | |
| 1 – 100 g per week | 183 | 76 (50) | 7 (23) | 0.005 | **0.23 (0.08-0.66)** | **0.006** |
| 101 – 200 g per week | 46 | 43 (12) | 3 (9) | 0.249 | 0.32 (0.04-2.71) | 0.296 |
| 201 – 350 g per week | 16 | 14 (4) | 2 (6) | 0.966 | 1.19 (0.16-9.05) | 0.865 |
| >350 g per week | 5 | 3 (1) | 2 (6) | 0.113 | 8.48 (0.95-75.48) | 0.055 |
| Beer (serves per week) (mean ± S.D.) | 385 | 0.32 ± 1.85 | 0.88 ± 3.16 | 0.209 |  |  |
| Wine (serves per week) (mean ± S.D.) | 384 | 3.39 ± 5.05 | 4.25 ± 10.57 | 0.446 |  |  |
| Spirits (serves per week) (mean ± S.D.) | 383 | 1.25 ± 3.43 | 0.97 ± 3.75 | 0.639 |  |  |
| Number of Pregnancies (mean ± S.D.) | 436 | 2.67 ± 1.61 | 1.94 ± 1.50 | 0.009 | 0.96 (0.69-1.34) | 0.821 |
| Pregnancy Age (years) (mean ± S.D.) | 373 | 25.68 ± 4.86 | 25.12 ± 5.23 | 0.578 |  |  |
| Hormone Replacement Therapy | 387 | 74 (21) | 3 (10) | 0.151 |  |  |

**Supplementary Table 4**: Female sub-analysis investigating the association between both WHO criteria I and III and characteristics/ lifestyle factors

| Characteristics | Sample Size | Controls (*N=*404) | SPS WHO Both Criteria (*N=*70) | Univariate Analysis | Multivariate Analysis | |
| --- | --- | --- | --- | --- | --- | --- |
|  |  | *N (%)* | *N (%)* | *­P-value* | *OR (95% CI)* | *P-value* |
| Age/ Diagnosis Age (years) (median, IQR) | 474 | 50 (43-54) | 34.5 (29-59) | <0.001 | 0.98 (0.94-1.01) | 0.210 |
| Affected with CRC | 474 | 0 (0) | 14 (20) |  | | |
| Smoking Status | 473 |  | | | | |
| Never Smoked | 240 | 204 (51) | 36 (52) | Ref. | | |
| Current Smoker | 81 | 74 (18) | 7 (10) | 0.152 | 0.52 (0.17-1.55) | 0.240 |
| Former Smoker | 152 | 126 (31) | 26 (38) | 0.578 | 1.72 (0.82-3.59) | 0.149 |
| Years Smoked (mean ± S.D.) | 469 | 9.08 ± 11.89 | 7.67 ± 12.95 | 0.366 | 0.99 (0.96-1.03) | 0.679 |
| Daily Cigarettes | 472 |  | | | | |
| No Cigarettes | 242 | 204 (51) | 38 (56) | Ref. | | |
| 1-5 Cigarettes per day | 52 | 38 (9) | 14 (21) | 0.058 | **2.72 (1.04-7.07)** | **0.040** |
| 6-10 Cigarettes per day | 62 | 53 (13) | 9 (13) | 0.818 | 1.24 (0.45-3.43) | 0.679 |
| > 10 Cigarettes per day | 116 | 109 (27) | 7 (10) | 0.013 | 0.33 (0.10-1.07) | 0.065 |
| Height (cm) (mean ± S.D.) | 426 | 162.54 ± 7.30 | 167.92 ± 8.60 | <0.001 | **1.11 (1.05-1.17)** | **<0.001** |
| Weight (kg) (mean ± S.D.) | 426 | 69.06 ± 16.00 | 71.27 ± 14.23 | - | | |
| Weight at 20 (kg) (mean ± S.D.) | 423 | 57.21 ± 11.01 | 63.13 ± 10.98 | - | | |
| BMI at registration (mean ± S.D.) | 422 | 26.17 ± 5.89 | 25.09 ± 4.47 | 0.149 | 0.99 (0.93-1.05) | 0.716 |
| BMI at 20 (mean ± S.D.) | 419 | 21.65 ± 3.73 | 22.28 ± 3.72 | 0.243 |  |  |
| Diabetes | 471 | 12 (3) | 4 (6) | 0.246 |  |  |
| Blood Lipid Lowering Medication | 459 | 20 (5) | 9 (14) | 0.016 | **12.62 (2.81-56.64)** | **0.001** |
| Aspirin (dose/week) (mean ± S.D.) | 469 | 0.76 ± 3.23 | 0.53 ± 2.23 | 0.544 |  |  |
| NSAIDs (dose/week) (mean ± S.D.) | 468 | 1.81 ± 4.82 | 0.55 ± 2.26 | 0.012 | 0.91 (0.79-1.06) | 0.243 |
| Antacids (dose/week) (mean ± S.D.) | 470 | 1.34 ± 5.34 | 0.58 ± 1.98 | 0.164 |  |  |
| Multivitamins (dose/week) (mean ± S.D.) | 470 | 1.97 ± 3.67 | 1.61 ± 3.18 | 0.435 |  |  |
| Calcium (dose/week) (mean ± S.D.) | 470 | 1.63 ± 3.23 | 1.19 ± 3.46 | 0.298 |  |  |
| Paracetamol (dose/week) (mean ± S.D.) | 469 | 2.14 ± 8.82 | 2.00 ± 6.56 | 0.903 |  |  |
| Folate (dose/week) (mean ± S.D.) | 463 | 1.83 ± 3.06 | 0.42 ± 1.67 | <0.001 | 0.85 (0.72-1.00) | 0.055 |
| Weekly Alcohol Consumption | 411 |  | | | | |
| No Alcohol | 137 | 115 (33) | 22 (37) | Ref. | | |
| 1 – 100 g per week | 202 | 176 (50) | 26 (43) | 0.410 | **0.38 (0.17-0.84)** | **0.017** |
| 101 – 200 g per week | 53 | 43 (12) | 10 (17) | 0.643 | 0.68 (0.20-2.27) | 0.527 |
| 201 – 350 g per week | 16 | 14 (4) | 2 (3) | 0.712 | 0.58 (0.06-5.73) | 0.638 |
| >350 g per week | 3 | 3 (1) | 0 (0) | - | 1 | - |
| Beer (serves per week) (mean ± S.D.) | 416 | 0.32 ± 1.85 | 0.53 ± 1.34 | 0.421 |  |  |
| Wine (serves per week) (mean ± S.D.) | 413 | 3.39 ± 5.05 | 2.44 ± 4.23 | 0.139 | **0.86 (0.75-0.98)** | **0.025** |
| Spirits (serves per week) (mean ± S.D.) | 415 | 1.25 ± 3.43 | 1.30 ± 4.58 | 0.921 |  |  |
| Number of Pregnancies (mean ± S.D.) | 468 | 2.67 ± 1.61 | 1.58 ± 1.53 | <0.001 | **0.75 (0.57-0.98)** | **0.035** |
| Pregnancy Age (years) (mean ± S.D.) | 391 | 25.68 ± 4.86 | 24.81 ± 4.26 | 0.260 |  |  |
| Hormone Replacement Therapy | 421 | 74 (21) | 5 (8) | 0.009 | 0.33 (0.08-1.32) | 0.116 |

**Supplementary Table 5**: Female sub-analysis investigating the association between CRC and characteristics/ lifestyle factors

| Characteristics | Sample Size | Controls (*N=*404) | SPS Cases with CRC (*N=*46) | Univariate Analysis | Multivariate Analysis | | |
| --- | --- | --- | --- | --- | --- | --- | --- |
|  |  | *N (%)* | *N (%)* | *­P-value* | *OR (95% CI)* | | *P-value* |
| Age/ Diagnosis Age (years) (median, IQR) | 448 | 50 (43-54) | 60 (38.5-71) | <0.001 | **1.06 (1.01-1.10)** | | **0.007** |
| Affected with CRC | 450 | 0 (0) | 46 (100) | - | | | |
| Smoking Status | 445 |  | | | | | |
| Never Smoked | 224 | 204 (51) | 20 (49) | Ref. | | | |
| Current Smoker | 77 | 74 (18) | 3 (7) | 0.164 | 0.20 (0.03-1.59) | 0.128 | |
| Former Smoker | 144 | 126 (31) | 18 (44) | 0.274 | 1.82 (0.84-3.95) | 0.131 | |
| Years Smoked (mean ± S.D.) | 442 | 9.08 ± 11.89 | 13.30 ± 18.05 | 0.053 | 1.02 (0.99-1.04) | | 0.248 |
| Daily Cigarettes | 443 |  | | | | | |
| No Cigarettes | 225 | 204 (51) | 21 (54) | Ref. | | | |
| 1-5 Cigarettes per day | 43 | 38 (9) | 5 (13) | 0.642 | 3.23 (0.97-10.79) | | 0.056 |
| 6-10 Cigarettes per day | 56 | 53 (13) | 3 (8) | 0.347 | 0.69 (0.19-2.59) | | 0.585 |
| > 10 Cigarettes per day | 119 | 109 (27) | 10 (25) | 0.775 | 0.83 (0.33-2.10) | | 0.690 |
| Height (cm) (mean ± S.D.) | 395 | 162.54 ± 7.30 | 164.26 ± 8.25 | 0.210 |  | |  |
| Weight (kg) (mean ± S.D.) | 395 | 69.06 ± 16.00 | 72.37 ± 13.91 | - | | | |
| Weight at 20 (kg) (mean ± S.D.) | 392 | 57.21 ± 11.01 | 58.34 ± 9.06 | - | | | |
| BMI at registration (mean ± S.D.) | 391 | 26.17 ± 5.89 | 26.70 ± 4.92 | 0.633 |  | |  |
| BMI at 20 (mean ± S.D.) | 388 | 21.65 ± 3.73 | 21.57 ± 2.71 | 0.908 |  | |  |
| Diabetes | 439 | 12 (3) | 2 (6) | 0.419 |  | |  |
| Blood Lipid Lowering Medication | 429 | 20 (5) | 6 (17) | 0.015 | 2.83 (0.83-9.71) | | 0.097 |
| Aspirin (dose/week) (mean ± S.D.) | 438 | 0.76 ± 3.23 | 0.80 ± 2.26 | 0.949 |  | |  |
| NSAIDs (dose/week) (mean ± S.D.) | 436 | 1.81 ± 4.82 | 0.23 ± 1.19 | 0.009 | 0.76 (0.58-1.01) | | 0.055 |
| Antacids (dose/week) (mean ± S.D.) | 439 | 1.34 ± 5.34 | 0.20 ± 1.18 | 0.065 | 0.89 (0.69-1.16) | | 0.393 |
| Multivitamins (dose/week) (mean ± S.D.) | 438 | 1.97 ± 3.67 | 1.09 ± 2.50 | 0.125 | 0.95 (0.83-1.09) | | 0.450 |
| Calcium (dose/week) (mean ± S.D.) | 437 | 1.63 ± 3.23 | 0.65 ± 2.01 | 0.052 | 0.88 (0.74-1.06) | | 0.175 |
| Paracetamol (dose/week) (mean ± S.D.) | 436 | 2.14 ± 8.82 | 2.91 ± 7.69 | 0.646 |  | |  |
| Folate (dose/week) (mean ± S.D.) | 431 | 1.83 ± 3.06 | 0.80 ± 2.26 | 0.037 | 0.93 (0.79-1.09) | | 0.361 |
| Weekly Alcohol Consumption | 383 |  | | | | | |
| No Alcohol | 122 | 115 (33) | 7 (22) | Ref. | | | |
| 1 – 100 g per week | 193 | 176 (50) | 17 (53) | 0.321 | 2.43 (0.81-7.26) | | 0.112 |
| 101 – 200 g per week | 50 | 43 (12) | 7 (22) | 0.081 | 3.28 (0.87-12.34) | | 0.079 |
| 201 – 350 g per week | 14 | 14 (4) | 0 (0) | - | 1 | | - |
| >350 g per week | 4 | 3 (1) | 1 (3) | 0.163 | 4.91 (0.39-62.47) | | 0.220 |
| Beer (serves per week) (mean ± S.D.) | 386 | 0.32 ± 1.85 | 0.24 ± 0.74 | 0.785 |  | |  |
| Wine (serves per week) (mean ± S.D.) | 384 | 3.39 ± 5.05 | 4.13 ± 5.71 | 0.456 |  | |  |
| Spirits (serves per week) (mean ± S.D.) | 385 | 1.25 ± 3.43 | 1.09 ± 3.78 | 0.789 |  | |  |
| Number of Pregnancies (mean ± S.D.) | 438 | 2.67 ± 1.61 | 2.74 ± 2.41 | 0.820 |  | |  |
| Pregnancy Age (years) (mean ± S.D.) | 379 | 25.68 ± 4.86 | 25.32 ± 4.98 | 0.695 |  | |  |
| Hormone Replacement Therapy | 390 | 74 (21) | 4 (13) | 0.244 |  | |  |

| Characteristics | Sample Size | Controls (*N=*404) | SPS Cases with No CRC (N=180) | Univariate Analysis | Multivariate Analysis | | |
| --- | --- | --- | --- | --- | --- | --- | --- |
|  |  | *N (%)* | *N (%)* | *­P-value* | *OR (95% CI)* | | *P-value* |
| Age/ Diagnosis Age (years) (median, IQR) | 580 | 50 (43-54) | 33.5 (28-50) | <0.001 | **0.94 (0.91-0.97)** | | **<0.001** |
| Affected with CRC | 584 | 0 (0) | 0 (0) | - | | | |
| Smoking Status | 579 |  | | | | | |
| Never Smoked | 301 | 204 (51) | 97 (55) | Ref. | | | |
| Current Smoker | 95 | 74 (18) | 21 (12) | 0.062 | 0.46 (0.21-1.01) | 0.054 | |
| Former Smoker | 183 | 126 (31) | 57 (33) | 0.805 | 1.10 (0.63-1.95) | 0.732 | |
| Years Smoked (mean ± S.D.) | 572 | 9.08 ± 11.89 | 7.51 ± 12.09 | 0.146 | 1.00 (0.98-1.02) | | 0.977 |
| Daily Cigarettes | 577 |  | | | | | |
| No Cigarettes | 305 | 204 (51) | 101 (58) |  | | | |
| 1-5 Cigarettes per day | 62 | 38 (9) | 24 (14) | 0.397 | 1.11 (0.50-2.46) | | 0.801 |
| 6-10 Cigarettes per day | 72 | 53 (13) | 19 (11) | 0.272 | 0.80 (0.36-1.76) | | 0.577 |
| > 10 Cigarettes per day | 138 | 109 (27) | 29 (17) | 0.010 | 0.59 (0.30-1.18) | | 0.139 |
| Height (cm) (mean ± S.D.) | 525 | 162.54 ± 7.30 | 167.29 ± 7.25 | <0.001 | **1.09 (1.05-1.13)** | | **<0.001** |
| Weight (kg) (mean ± S.D.) | 525 | 69.06 ± 16.00 | 72.41 ± 15.09 |  | | | |
| Weight at 20 (kg) (mean ± S.D.) | 517 | 57.21 ± 11.01 | 65.60 ± 14.90 |  | | | |
| BMI at registration (mean ± S.D.) | 520 | 26.17 ± 5.89 | 25.95 ± 5.73 | 0.690 |  | |  |
| BMI at 20 (mean ± S.D.) | 514 | 21.65 ± 3.73 | 23.59 ± 5.78 | <0.001 | **1.09 (1.03-1.16)** | | **0.003** |
| Diabetes | 573 | 12 (3) | 6 (4) | 0.720 |  | |  |
| Blood Lipid Lowering Medication | 559 | 20 (5) | 18 (11) | 0.016 | **8.94 (2.94-27.18)** | | **<0.001** |
| Aspirin (dose/week) (mean ± S.D.) | 570 | 0.76 ± 3.23 | 0.23 ± 1.45 | 0.016 | 0.93 (0.81-1.06) | | 0.252 |
| NSAIDs (dose/week) (mean ± S.D.) | 569 | 1.81 ± 4.82 | 0.56 ± 2.53 | <0.001 | 0.94 (0.86-1.02) | | 0.111 |
| Antacids (dose/week) (mean ± S.D.) | 571 | 1.34 ± 5.34 | 0.71 ± 2.33 | 0.099 | 0.99 (0.90-1.08) | | 0.757 |
| Multivitamins (dose/week) (mean ± S.D.) | 571 | 1.97 ± 3.67 | 1.54 ± 3.01 | 0.163 |  | |  |
| Calcium (dose/week) (mean ± S.D.) | 571 | 1.63 ± 3.23 | 0.74 ± 2.56 | 0.001 | 1.00 (0.90-1.10) | | 0.975 |
| Paracetamol (dose/week) (mean ± S.D.) | 569 | 2.14 ± 8.82 | 1.49 ± 5.17 | 0.354 |  | |  |
| Folate (dose/week) (mean ± S.D.) | 564 | 1.83 ± 3.06 | 0.40 ± 1.79 | <0.001 | **0.82 (0.73-0.92)** | | **0.001** |
| Weekly Alcohol Consumption | 502 |  |  |  |  | |  |
| No Alcohol | 182 | 115 (33) | 67 (44) |  |  | |  |
| 1 – 100 g per week | 233 | 176 (50) | 57 (38) | 0.007 | **0.33 (0.18-0.60)** | | **<0.001** |
| 101 – 200 g per week | 63 | 43 (12) | 20 (13) | 0.469 | 1.09 (0.46-2.60) | | 0.842 |
| 201 – 350 g per week | 20 | 14 (4) | 6 (4) | 0.548 | 0.75 (0.17-3.22) | | 0.697 |
| >350 g per week | 4 | 3 (1) | 1 (1) | 0.632 | 0.67 (0.053-8.39) | | 0.755 |
| Beer (serves per week) (mean ± S.D.) | 514 | 0.32 ± 1.85 | 0.61 ± 1.99 | 0.112 | 1.00 (0.86-1.17) | | 0.965 |
| Wine (serves per week) (mean ± S.D.) | 506 | 3.39 ± 5.05 | 2.61 ± 5.78 | 0.108 | 1.00 (0.95-1.05) | | 0.925 |
| Spirits (serves per week) (mean ± S.D.) | 512 | 1.25 ± 3.43 | 0.90 ± 3.27 | 0.256 |  | |  |
| Number of Pregnancies (mean ± S.D.) | 567 | 2.67 ± 1.61 | 1.60 ± 1.51 | <0.001 | 0.85 (0.70-1.02) | | 0.084 |
| Pregnancy Age (years) (mean ± S.D.) | 457 | 25.68 ± 4.86 | 25.50 ± 5.11 | 0.734 |  | |  |
| Hormone Replacement Therapy | 514 | 74 (21) | 10 (6) | <0.001 | 0.51 (0.19-1.35) | | 0.175 |

**Supplementary Table 6**: Female sub-analysis investigating the association between SPS patients with no CRC and characteristics/ lifestyle factors
